# Cathepsins in Neurological Diseases

**DOI:** 10.3390/ijms26167886

**Published:** 2025-08-15

**Authors:** Dominik Lewandowski, Mateusz Konieczny, Agata Różycka, Krzysztof Chrzanowski, Wojciech Owecki, Jan Kalinowski, Mikołaj Stepura, Paweł Jagodziński, Jolanta Dorszewska

**Affiliations:** 1 Laboratory of Neurobiology, Department of Neurology, Poznan University of Medical Sciences, 61-701 Poznan, Poland; dlewandowski237@gmail.com (D.L.); konieczny.mateusz788@gmail.com (M.K.); 91198@student.ump.edu.pl (K.C.); 86897@student.ump.edu.pl (W.O.); 86089@student.ump.edu.pl (J.K.); 82950@student.ump.edu.pl (M.S.); 2 Department of Biochemistry and Molecular Biology, Poznan University of Medical Sciences, 61-701 Poznan, Poland; arozycka@ump.edu.pl (A.R.); pjagodzi@ump.edu.pl (P.J.)

**Keywords:** neurodegenerative diseases, cathepsins, neuroinflammation, proteolysis, autophagy

## Abstract

Cathepsins, a family of lysosomal proteases, play critical roles in maintaining cellular homeostasis through protein degradation and modulation of immune responses. In the central nervous system (CNS), their functions extend beyond classical proteolysis, influencing neuroinflammation, synaptic remodeling, and neurodegeneration. Emerging evidence underscores the crucial role of microglial cathepsins in the pathophysiology of several neurological disorders. This review synthesizes current knowledge on the involvement of cathepsins in a spectrum of CNS diseases, including Parkinson’s disease, Alzheimer’s disease, multiple sclerosis, amyotrophic lateral sclerosis, epilepsy, Huntington’s disease, and ischemic stroke. We highlight how specific cathepsins contribute to disease progression by modulating key pathological processes such as α-synuclein and amyloid-β clearance, tau degradation, lysosomal dysfunction, neuroinflammation, and demyelination. Notably, several cathepsins demonstrate both neuroprotective and pathogenic roles depending on disease context and expression levels. Additionally, the balance between cathepsins and their endogenous inhibitors, such as cystatins, emerges as a critical factor in CNS pathology. While cathepsins represent promising biomarkers and therapeutic targets, significant gaps remain in our understanding of their mechanistic roles across diseases. Future studies focusing on their regulation, substrate specificity, and interplay with genetic and epigenetic factors may yield novel strategies for early diagnosis and disease-modifying treatments in neurology.

## 1. Introduction

Microglia, the resident macrophages of the central nervous system (CNS), play a pivotal role in maintaining neuronal homeostasis and orchestrating inflammatory responses within the brain. As key components of the innate immune system, microglia continuously survey the CNS microenvironment, responding to injury, infection, and pathological changes by modulating inflammation and clearing cellular debris. While a tightly regulated inflammatory response is essential for CNS health and repair, prolonged or dysregulated inflammation can have deleterious consequences, contributing to the onset and progression of various neurodegenerative diseases [1].

**Figure 1:**
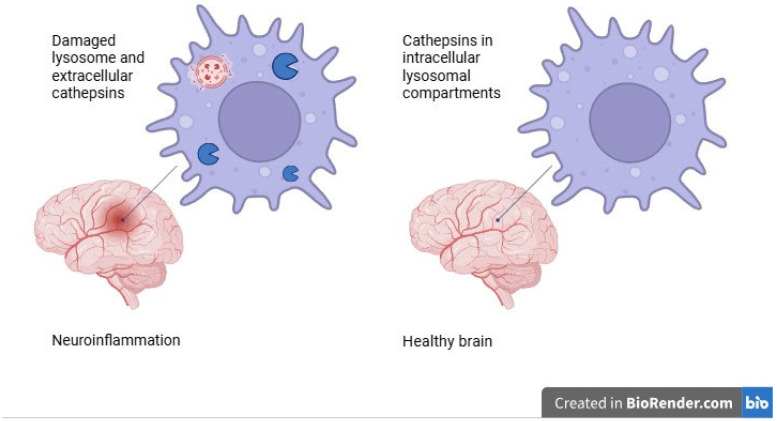
Impact of cathepsins’ localization on neuroinflammation.

However, many aspects of this interplay remain poorly understood. One critical area of investigation is the role of microglia and their secreted mediators in modulating neuroinflammatory and neurodegenerative processes [2]. Among these mediators, cathepsins—lysosomal proteases involved in protein degradation and cellular turnover—have emerged as important regulators of both physiological and pathological pathways in the CNS. Cathepsins comprise a broad group of proteolytic enzymes that include 11 human cysteine proteases (B, C, F, H, K, L, O, S, V, W, X), two aspartic proteases (D and E), and two serine proteases (A and G). These enzymes are typically active in mildly acidic environments and were originally considered to be confined to intracellular lysosomal compartments. However, it is now evident that under pathological conditions, cathepsins can also function extracellularly, contributing to the degradation of the extracellular matrix, potential mechanism of its action is presented in Figure 1. Moreover, active cathepsins have been identified in various subcellular locations such as the nucleus, plasma membrane, and cytoplasm, indicating a broader scope of activity than previously understood [3,4].

## 2. Materials and Methods

For this review, the relevant literature was identified through comprehensive searches of the electronic databases, PubMed, ScienceDirect, and Web of Science, with an emphasis on the most up-to-date research. The search strategy employed key terms such as “neuroinflammation”, “inflammatory”, “parkinson’s disease”, “alzheimer’s disease”, “neurodegenerative disease”, “stroke”, “multiple sclerosis”, “epilepsy”, “huntington’s disease”, “amyotrophic lateral sclerosis”, and “cathepsins”. Duplicates were excluded, and relevant studies were selected and refined according to their relevance and quality for inclusion in this review. Inclusion criteria for this review were experimental (in vitro and in vivo) and human studies; studies published in English; and studies addressing molecular, pathological or therapeutic significance of cathepsins. Exclusion criteria for this article were studies without specific reference to CNS, articles not available in English language, and reviews without original data (used in the article only to provide background information). The selection process was conducted by four independent researchers, and all disagreements were resolved through discussion. During the identification stage, 2652 records were obtained from databases (57—ScienceDirect; 2595—PubMed). After eliminating duplicates, 2325 records were subjected to screening, and out of these, 2036 were excluded due to irrelevant topics. The remaining 289 papers underwent further scrutiny, and, finally, a total of 100 papers were included in the current review.

## 3. Cathepsins in Neuroinflammation and Aging

Cathepsin D serves as the principal endopeptidase involved in the degradation of long-lived proteins, including α-synuclein—a process relevant to neurodegenerative diseases such as Parkinson’s disease (PD) and dementia with Lewy bodies (DLB). Cathepsin D facilitates α-synuclein clearance, although its overexpression in the primate models with PD resulted in neuronal death [5,6]. Several cysteine proteases also play essential roles in CNS function. Cathepsin H present in perivascular microglia modulates immune responses. In vitro studies have shown that inflammatory stimulation enhances cathepsin H release and activity in microglia, leading to increased IL-1β and IFN-γ production, accompanied by neuronal damage [7]. Cathepsins B and L are implicated in both intracellular proteolysis and extracellular matrix remodeling. Their deficiency is lethal in early development in mice models [8]. Additionally, their role in intracellular cholesterol trafficking has been highlighted, with inhibition of these enzymes inducing changes akin to Niemann–Pick type C disease through NPC1 and ABCA1 dysregulation [9]. Nonetheless, a 2002 study revealed that inhibition of cathepsins B and L in aged rat hippocampal slices led to a compensatory increase in cathepsin D levels. Age-related upregulation of cathepsin D has also been observed in rat brains and in the cerebrospinal fluid of patients with Alzheimer’s disease (AD), suggesting its potential involvement in neurodegenerative processes [10]. Cathepsin X is a carboxypeptidase found in immune cells. Its upregulation has been documented in lipopolysaccharide (LPS)-induced inflammation. Inhibition of cathepsin X with the specific inhibitor AMS36 significantly reduced LPS-induced oxidative and inflammatory responses. These findings suggest that cathepsin X may be a therapeutic target for mitigating microglia-induced neurotoxicity in neuroinflammatory conditions [11].

Aging is another critical factor influencing neuroinflammatory processes. Aged microglia show reduced phagocytosis and increased senescence markers [12]. Cathepsin C, which is mainly peripheral, is present at low levels in the brain. It plays a crucial role in activating serine proteases within cytotoxic T cells, natural killer cells, mast cells, and neutrophils. Following LPS-induced inflammation, cathepsin C expression was observed in microglia throughout the brain, though its precise role in neuroinflammation warrants further investigation [13]. Notably, previous studies have shown that the inhibition of cathepsin C exacerbated demyelination in the cuprizone model [14]. Cystatin C, a key endogenous inhibitor of cathepsins, also contributes significantly to demyelination dynamics. In multiple sclerosis (MS), increased levels of cathepsins have been detected in microglia within white matter lesions, while elevated cystatin C expression has been noted in astrocytes. A shift toward cathepsin activity can impair remyelination [15]. Cathepsin S remains active outside of the lysosomes. Its elevated levels have been observed in the CNS of aged mice and in the spinal cord of ALS transgenic mouse models, suggesting its involvement in disease pathogenesis. However, additional studies are needed to clarify its specific contributions to neurodegeneration [16]. Cathepsin K, although best known for its role in bone resorption, is also present in brain parenchyma, particularly the choroid plexus. Its deficiency induced behavioral and cognitive changes in mice [17]. Cathepsins play multifaceted roles in the CNS, contributing to neuroinflammation, proteostasis, and cellular remodeling; key characteristics and associations are presented in Table 1. Their dysregulation is increasingly linked to neurodegenerative and demyelinating diseases, with both protective and pathogenic effects depending on context and compartment. Notably, interactions between cathepsins and endogenous inhibitors like cystatin C appear to influence disease progression, particularly in disorders such as multiple sclerosis and AD. Although clinical translation remains limited, current evidence supports their potential as dynamic biomarkers and therapeutic targets. Future research should focus on isoform-specific modulation and improved CNS-targeted delivery to advance cathepsin-based strategies in clinical application.

## 4. Cathepsins and Parkinson’s Disease

PD is the second most common neurodegenerative disorder, with a prognosis of increasing prevalence [18]. The Global Burden of Disease Study indicates that in 2021, 11.77 million people were affected by PD worldwide, with a higher burden of PD among males than in females [19]. The recent estimates suggest that this number will increase to 25.2 million PD patients globally in 2050 [20]. Furthermore, there are no available disease-modifying pharmacologic treatment approaches for PD. The treatment is symptomatic, aiming to alleviate both motor and nonmotor symptoms [21,22,23]. The etiology of PD involves genetic, behavioral, and environmental factors [24]. Pathologically, PD is characterized by deposition of aggregated proteins in Lewy neurites and Lewy bodies, as well as nigral dopaminergic neurodegeneration [25]. In this context, the major hallmark of PD is the accumulation of α-synuclein aggregates [26]. Evidence shows that cathepsins are also implicated in PD pathogenesis [27].

In recent years, studies have demonstrated the involvement of the autophagy lysosomal pathway in PD etiology, and cathepsins are implicated in the lysosomal degradation of α-synuclein [28,29]. Cathepsins B and L cleave within the circumvent fibril formation and α-synuclein amyloid region. Cathepsin D requires the presence of anionic phospholipids to degrade α-synuclein [28]. Increasing cathepsin D activity not only intensifies α-synuclein degradation but also restores autophagy and endo-lysosome functions [30]. Studies report inconsistent cathepsin D levels in PD patients, with findings of both elevation and reduction in plasma and CSF [31,32,33]. On the other hand, cathepsin L was overexpressed in dopamine neurons of postmortem PD brains analyzed by immunofluorescent staining [34]. In addition, recent evidence, based on genome-wide association studies (GWAS) analysis, revealed that increased levels of cathepsin B are associated with lower PD risk, whereas no significant associations were found for cathepsin E, F, G, H, L1, L2, O, S, and Z [27,35]. In mice models, deficiency of cathepsins B and L was linked with brain atrophy, neurodegeneration, and reactive astrocytosis [8]. Interestingly, the protective effect of cathepsin B seems to be mediated by N-acetylaspartate [36]. Interestingly, recent studies revealed a dual role of cathepsin B, which, firstly, promoted amyloid degradation (reducing its toxicity) while maintaining ordered morphology, and, secondly, altered the regularity of the secondary and tertiary structure of amyloid, making these structures more flexible [37,38]. The authors indicated that cathepsin B inhibition disturbs autophagy, hinders pre-formed α-synuclein fibrils clearance, impairs glucocerebrosidase function, and contributes to lysosomal content deposition [38]. Concomitantly, cathepsin D may also exhibit neuroprotective effects in PD, reducing α-synuclein toxicity via an interplay with calcineurin. Cathepsin D requires functional calcineurin signaling to maintain proper vacuolar proteolytic function and pH homeostasis, enabling the reduction in α-synuclein oligomers [39]. On the other hand, α-synuclein directly hinders the enzymatic functions of cathepsins, disrupting lysosomal trafficking of cathepsin B, cathepsin D, and cathepsin L. This phenomenon decreases the proteolytic activity of cathepsins, reducing α-synuclein clearance [40].

On the other hand, the cathepsins’ involvement in PD may not be clearly observed. Mantle et al. [41] investigated the activity of cathepsins B, D, H, and L in the frontal cortex tissue of PD patients, with no significant differences compared with healthy controls. In contrast, another study analyzed postmortem late-stage PD temporal cortex specimens and revealed a significant decrease in activity for cathepsin D, with no considerable alterations in cathepsin B [42]. Concomitantly, cathepsin D activity decrease was also described in substantia nigra of PD patients [43]. In addition, there are reports suggesting no significant alterations of cathepsin D in the plasma of PD patients [44]. Moreover, a 2003 published study failed to confirm a direct association between cathepsin D genotype and PD [45]. In contrast, a recent study suggests that differences in cathepsin levels may be associated with particular genetic PD etiology. Cathepsin D levels measured in macrophages derived from peripheral blood mononuclear cells differed among PD patients with *LRRK2* mutation and with *GBA-1* mutation, indicating distinct autophagy dynamics linked with genetic mutations [46]. Genetic mutations such as *LRRK2* G2019S may promote α-synuclein aggregation, and decrease cathepsin B and L activities [47]. Cathepsin-mediated α-synuclein truncation may enhance aggregation propensity and promote fibril formation [48,49,50]. McGlinchey et al. [50] suggest that the enrichment of aggregation-prone α-synuclein truncations appears due to an imbalance between α-synuclein and cathepsin levels in the lysosome, causing incomplete α-synuclein degradation. Moreover, another study revealed that knockdown of cathepsin B decreased fibril-induced aggregates formation, suggesting that cathepsin B may trigger intracellular α-synuclein aggregates formation [51]. On the other hand, studies show that cathepsins B and L are prone to oxidation from reactive carbonyls, causing their inactivation. This process may occur if there is a high abundance of oxidants, favoring cathepsin D, which is not susceptible to this phenomenon. Concomitantly, cathepsin D induces α-synuclein truncations oligomerization and downstream pore formation, whereas cathepsins B and L oppose this process. In consequence, the likelihood of downstream aggregation increases [28,52]. Additionally, cathepsin B may impact the synthesis and release of interleukin-1β (IL-1β) by pyrin domain-containing protein 3 inflammasome-independent processing of procaspase-3 [53]. Cathepsin B has been implicated in IL-1β release through nod-like receptor protein 3 (NLRP3) inflammasome-independent pathways, contributing to α-synuclein-induced neuroinflammation [54,55]. On the other hand, Pišlar et al. in a series of articles [56,57,58] conclude that cathepsin X may be considered a pathogenic factor in PD. For instance, in a PD mouse model, cathepsin X was upregulated in the injured dopaminergic system, suggesting its involvement in PD pathology [56]. Upregulation of cathepsin X in PD models promotes microglial activation and neurodegeneration, whereas its inhibition appears neuroprotective [11,57]. Similarly, Gan et al. [58] demonstrated that the knockdown of cathepsin D contributed to a decrease in inflammation-mediated dopaminergic neurodegeneration via inhibition of the NF-κB signaling pathway in a PD mouse model. Furthermore, cathepsin L inhibition in PD dopaminergic neurons restored decreased glucocerebrosidase levels and diminished phosphorylated α-synuclein burden [59]. GWAS data suggest cathepsin B may be protective, while cathepsins H and S are associated with higher PD risk [60]. In contrast, another study observed a causal relationship between cathepsin B and cathepsin D with PD [61]. To sum up, the role of cathepsins in PD remains controversial since both protective and pathogenic effects are described; theinfluence of cathepsins on disease pathology is shown in Table 2. Cathepsins B, D, and L appear to be the most extensively studied. Considering that studies report diverse influences of cathepsins in PD pathogenesis, it is crucial to investigate their potential utility as therapeutic targets in a particular context, with regard to their neuroprotective or neurodegenerative effects. For instance, the development of specific cathepsin inhibitors, activated under selected conditions, may contribute to the alleviation of PD pathology. Moreover, enhancing cathepsin activity associated with neuroprotective outcomes constitutes another potential strategy of reducing PD-associated neurodegeneration, and further research is necessary to fully elucidate their implication in PD pathogenesis.

## 5. Cathepsins and Alzheimer’s Disease

**Figure 2:**
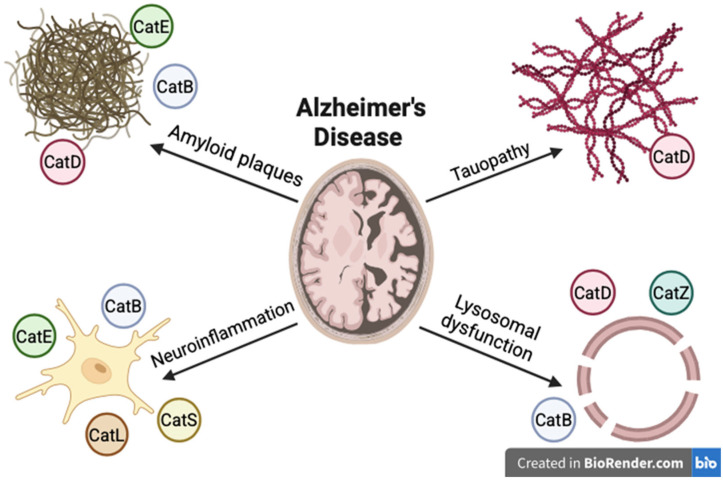
Alzheimer’s disease pathophysiology.

AD is the most common neurodegenerative process and the most common cause of dementia. AD is a growing global health burden, with its incidence expected to triple by 2050 [62]. The molecular basis of the disease is the deposition of senile plaques, extracellular aggregates of amyloid β (Aβ), and the intracellular accumulation of hyperphosphorylated tau (pTau) in the form of neurofibrillary tangles. This process leads to progressive neurodegeneration and cognitive decline [63]. The risk of the disease depends largely on genetic factors, with *APOE* being the most strongly associated with AD [62]. Additionally, aging, chronic inflammation, environmental factors, and lifestyle play an important role [64]. Despite advanced molecular research, the mechanisms of the disease are not fully understood, and the lack of effective causal therapies reflects its complexity. The paucity of effective therapies underscores not only the complexity of AD pathogenesis, but also the pressing need for an interdisciplinary approach that integrates biochemistry, genetics, and neuroimmunology. Cathepsin D degrades Aβ and tau linking it to AD pathogenesis. Increased expression of cathepsin D may represent an adaptive response to the processes leading to neurofibrillary degeneration in AD [65]. However, it is worth considering that alterations in cathepsin D expression in AD may modulate alternative neurodegenerative pathways, independently of classical disease markers. Histochemical analyses of brain tissue indicate that senile plaques arise from mixtures of Aβ and cathepsin D released during vascular hemolysis and microaneurysm rupture, underlining the involvement of cathepsin D in amyloid pathology [66]. Functionally, cathepsin D degrades both Aβ and tau in vitro, and studies in mouse models have shown that the absence of CatD leads to dramatic accumulation of Aβ in lysosomes, where Aβ and tau are normally transported and degraded. Interestingly, the Aβ42 peptide, the most amyloidogenic Aβ species, is a potent competitive inhibitor of cathepsin D, which may impair the degradation of pathological forms of tau and promote tauopathy in AD [67,68]. Serum cathepsin D levels have been associated with AD dementia and cerebral atrophy, suggesting the potential of cathepsin D as a prognostic biomarker of global cognitive and functional decline [69]. Plasma cathepsin D levels were lower in individuals with amyloid plaques in the brain compared to controls, and a logistic regression model indicates a high efficacy of plasma cathepsin D as a diagnostic biomarker for AD [70]. The genetic polymorphism C ⇒ T in the CATD gene, encoding cathepsin D, is associated with a significant but small increase in the risk of AD in the Caucasian group, especially in the presence of the *APOE ε*4 allele [71,72,73]. Furthermore, studies of skin fibroblasts from AD patients have shown reduced CATD expression at the transcriptional and translational level and changes in its processing, which may be regulated by the Ras oncogene and the p38 MAPK pathway. This suggests systemic cathepsin D dysfunction in the pathogenesis of AD [74]. It is important to emphasize that these observations may carry broader practical implications, particularly in the search for biomarkers detectable outside the central nervous system. Cathepsin B is a cysteine protease, a key enzyme for the proteolytic degradation of amyloid precursor protein (APP) in the brain. This prevents the accumulation of pathological aggregates of Aβ and the formation of plaques characteristic of AD [75]. In animal models and in vitro, cathepsin B has been shown to cleave amyloid β, especially the Aβ1-42 form, reducing its aggregation and amyloid deposits, which indicates its neuroprotective function. Genetic inactivation of cathepsin B leads to increased Aβ deposition and the exacerbation of AD pathology, while increased cathepsin B expression reduces amyloid deposits [76]. Of particular interest is the potential of these findings, especially with regard to the prospective use of cathepsin B as a therapeutic target in clinical settings. Clinical studies have shown elevated levels of cathepsin B in the plasma of AD patients compared to healthy controls, but CSF levels of this enzyme do not show significant differences between groups [77]. Cystatin B (CstB), encoded on chromosome 21, is an endogenous inhibitor of cathepsin B. Down syndrome patients show increased expression of CstB, which correlates with early-onset AD risk [78]. In addition, cathepsin B plays an important role in microglial clearance of Aβ, modulating microglial phagocytic functions via activation of the PI3K-AKT signaling pathway. Cathepsin B deficiency impairs Aβ clearance efficiency and cognitive function in mice, confirming its importance in the pathophysiology of AD [79]. At the same time, lysosomal leakage is observed in AD, in which cathepsin B redistributed to the cytosol initiates neurodegenerative processes such as apoptosis and inflammation. Cathepsin E is an aspartic protease that modulates microglial activation and neurodegeneration in AD. It promotes Aβ accumulation and neuroinflammation. In animal models, ablation of cathepsin E led to reductions in Aβ accumulation, neuroinflammation, and cognitive impairment. In mice with AD, administration of cathepsin E inhibitors reduced neuroinflammation and Aβ accumulation, ultimately restoring memory function. These data suggest that cathepsin E may be considered a potential therapeutic target for AD [80,81]. Cathepsin L contributes to the nuclear membrane damage. Evidence suggests that increased expression of cathepsin L induces the cleavage of lamin B1. This process can be regulated by pharmacological or genetic suppression of cathepsin L, which alleviates the degradation of lamin B1 induced by the Aβ42 peptide and the associated structural and molecular changes [82]. In addition, cathepsin L may affect the immunopathogenesis of AD through its role in the degradation of the invariant chain (Ii), which blocks the antigen-binding site of the class II histocompatibility complex. Activation of microglia initially induces the expression of Ii, and then the progression of microglia activation and proliferation in the AD brain leads to the degradation of the Ii chain by pcathepsin L [83]. Cathepsin S is a cysteine protease implicated in inflammation in many diseases. Increased levels of cathepsin S have been reported in the serum of elderly individuals. In a mouse model, an increase in cathepsin S expression in hippocampal neurons associated with aging was demonstrated, which resulted in a decline in recognition function. Overexpression of cathepsin S in neurons enhanced the neuroinflammatory environment, activating microglia to a proinflammatory M1 phenotype and the CX3CL1-CX3CR1 and JAK2-STAT3 pathways, revealing a role for cathepsin S in neuron–microglia communication. Increased expression of cathepsin S in the brain, especially in the hippocampus, was reported in AD patients. A CatS inhibitor (LY3000328) alleviated AD symptoms in mice. Overexpression of cathepsin S increased cathepsin B activity and decreased cathepsin L activity in microglia. CatS may be a potential biomarker of AD [84]. Therapeutically, the concentration of cathepsins may be influenced by urolithin A (UA), a metabolite of ellagic acid in the intestine. UA stimulates mitophagy and may regulate cathepsin Z, a potential AD target [85]. The role of cathepsins—including their effects on amyloid, tau, neuroinflammation, and lysosomal function—is a key and multifaceted element of AD pathogenesis. The main mechanisms were presented in Figure 2.

## 6. Cathepsins and Stroke

Stroke is an episode of acute and persistent neurological dysfunction caused by cerebral ischemia or hemorrhage. It is the second-leading cause of death worldwide and poses a significant threat due to its high morbidity, mortality, and rate of recurrence. Over the 20-year period from 1990 to 2019, the incidence rate of stroke has increased by 70%. Despite its severity, most strokes are preventable through the control of modifiable risk factors. Recurrence of stroke worsens patients’ outcomes and increases the healthcare burden [86]. Liu et al. have reported cathepsin C to aggravate neuroinflammation and mediate neurological injury by neurotoxic polarization of microglia [87]. As research shows, cathepsin C and cathepsin S are involved in various mechanisms of cell pathology, with elevation in levels of both proteins found in aneurysmal subarachnoid hemorrhage suggesting their involvement in developing neuronal damage [88]. Cathepsin S contributes to neuroinflammation and blood–brain barrier breakdown in ischemic stroke and subarachnoid hemorrhage. Its elevated serum levels may serve as a biomarker, and inhibition improves outcomes in pre-clinical models [89,90,91,92]. These findings suggest that cathepsin C and cathepsin S could serve as promising biomarkers in understanding secondary pathological injury and potential drug targets following post-subarachnoid hemorrhage [87]. In stroke patients cathepsin S has been shown to exacerbate neuroinflammation, microglial activation, and neuronal injury, with research pointing to potential benefits associated with decreasing its activity following cerebrovascular incident. For the time being, a need for further clinical studies and exploration of cathepsin S inhibitors safety in human trial is required. The diagnostic potential of both cathepsin C and S might provide useful in screening stroke patients if changes pre-incident are shown to bring prognostic value, which remains to be proven. Cathepsin B and K are overexpressed in cerebral aneurysms and may promote rupture risk [93]. Conversely, recombinant tissue plasminogen activator treatment following knockout of cathepsin K was shown to increase the severity and worsen the neurological outcome of hemorrhagic transformation, a common complication for this [94]. Cathepsin B contributes to ferroptosis after hemorrhage and its inhibition improves microglial survival [95]. Importantly, there are several proofs that cathepsins, associated with stroke level, can be elevated in individuals with well-known risk factors before stroke events. Cathepsin S is elevated in people with atherosclerosis, hypertension, obesity and insulin resistance; cathepsin D baseline level is increased in smokers and individuals with diabetes and there is proof that it is associated with future coronary events; cathepsin K level is elevated in patients with atherosclerotic risk; however, there is no study linking it directly to subsequent stroke [96,97,98].

A study using Mendelian randomization conducted by Sun et al. found that elevated levels of cathepsin E and O are associated with increased ischemic stroke risk [61]. Serum cathepsin G levels were found to correlate with D-dimer concentrations and were significantly associated with both arterial and venous thrombosis. Although this elevation was not identified as an independent risk factor for stroke within this study, increased thrombotic activity constitutes an underlying risk milieu conducive to cerebrovascular events [99]. Restoring CatD function post-stroke may protect neurons from hypoxia-induced lysosomal damage [100]. Considering emerging research concerning cathepsins in stroke, they represent potential biomarkers and therapeutic targets that require further human studies to clarify their usage and place in the diagnosis and treatment of cerebrovascular incidences. As most data is based upon preclinical models, small observational cohorts, or indirect genetic associations, limiting the strength of causal inference, findings should be interpreted with caution until evidence from large, long-term human studies is obtained. The current significance of cathepsins in stroke management is presented in Table 3.

Cathepsin-A-related arteriopathy in strokes and leukoencephalopathy (CARASAL) is a rare, adult-onset autosomal-dominant hereditary disease, affecting small cerebral vessels. It is associated with the c.973C > T variant in *CTSA* gene, located on chromosome 20q13.12. The *CTSA* encodes cathepsin A, a serine carboxypeptidase of the peptidase S10 family, which is responsible for a variety of functions, including stabilization of beta-galactosidase and neuraminidase, as well as degradation of endothelin-1. Clinical manifestations of CARASAL are dominated by ischemic and hemorrhagic strokes, as well as TIAs occurring between the third and fifth decade. A broad spectrum of accompanying symptoms has been reported, including gradual cognitive decline, headaches, migraines, vertigo, tinnitus, sensorineural hearing loss restless legs syndrome, gait disturbance, and depression. Notably, symptoms may be minimal or absent despite the presence of extensive leukoencephalopathy. Cerebral MRI typically reveals diffuse leukoencephalopathy, involving the white matter of the brainstem (including pyramidal tracts, tegmental tracts, middle, and superior cerebellar peduncles) and subcortical white matter, with sparing of the U-fibers. Regions of gray matter within the thalamus, the basal ganglia, and the right dentate nucleus have been reported to be impacted. Hyperintensities within the white matter are hypothesized to be the result of elevated endothelin-1 levels, which inhibit the maturation of oligodendrocyte progenitor cells, thus disturbing proper myelination [101,102]. To date, recommended specific treatment in CARASAL has not been established with no evidence for thrombolysis, antithrombotic or anticoagulation treatment being indicated [103]. However, gene therapy has been shown promising in cell models of the disease. As of now, 19 patients have been reported, but the frequency of CARASAL diagnosis is expected to rise as genetic testing becomes more widely accessible [104]. Investigating the contribution of cathepsin A to stroke risk in the general population could provide a promising frontier as the prevalence of CARASAL remains largely unknown due to the limited availability of its diagnostic.

## 7. Cathepsins and Multiple Sclerosis

Multiple sclerosis (MS) is a chronic autoimmune disease of the CNS, characterized by disseminated demyelination and neuroinflammation [105]. The pathogenesis of MS involves a complex interplay between genetic and environmental factors, many of which are not yet fully understood. Key risk factors include Epstein–Barr virus infection, vitamin D deficiency, smoking, and genetic predispositions linked to the major histocompatibility complex (HLA) [106,107].

**Figure 3:**
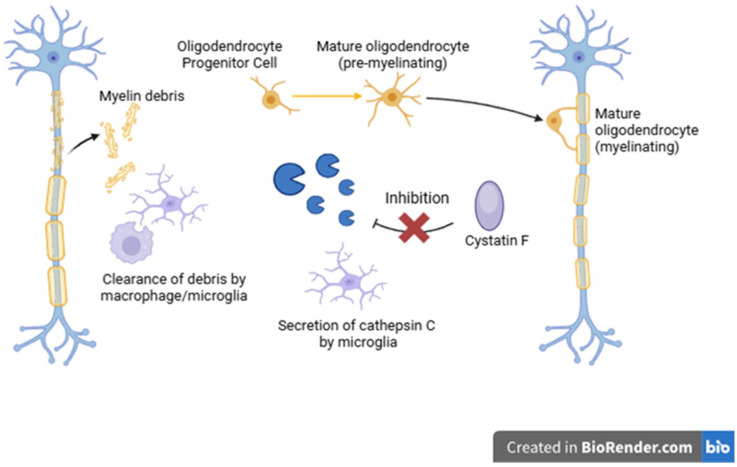
Potential mechanism of remyelination.

MS is now therapeutically modifiable due to improved understanding and disease-modifying therapeutics [108]. Nonetheless, many patients still experience gradual progression of disability, often independent of relapses, suggesting the additional pathological mechanisms [109]. In response to the increasing interest in the molecular mechanisms underlying disease progression, lysosomal proteases—particularly cathepsins—have gained attention. Their roles in regulating inflammatory processes, tissue remodeling, and neurodegeneration may be key to better understanding both the pathogenesis and potential therapeutic targets in MS. Cathepsins regulate inflammation, apoptosis, and ECM remodeling, implicating them in MS pathogenesis [110,111]. Cathepsin B, participating in the degradation of cytoskeletal proteins and myelin sheaths, was particularly increased in active inflammatory lesions. Moreover, the intensity of demyelinating and neurotoxic processes significantly correlated with elevated cathepsin B activity [61,112]. Similar conclusions were drawn from studies on cathepsin S, which is involved in the degradation of MHC class II chains, placing it as a regulator of immune function through the modulation of CD4+ T lymphocyte activity [111]. An intriguing mechanism has also been proposed for cathepsin X/Z, which acts as a regulator of cell adhesion and lymphocyte migration. In the experimental autoimmune encephalomyelitis (EAE) model, the inhibition of this protease resulted in clinical symptom alleviation and a reduction in inflammatory infiltrates in the brain [113]. Importantly, cathepsin Z activity is thought to be modifiable through molecular engineering, which grants it additional value as both a potential biomarker and therapeutic target [114]. Another significant finding is the relationship between increased cathepsin activity and decreased levels of their natural inhibitors—cystatins. Disruptions in this balance have been observed in the peripheral blood serum of MS patients and animal models, where the degree of variation between these two factors correlated with inflammatory response intensity and the progression of CNS demyelination. It is anticipated that with further advancements in molecular engineering, targeted molecular therapies could restore homeostatic balance in the cathepsin-cystatin axis, effectively reducing disease activity [15,112,114]. Cystatin F induction ceased in chronic demyelination when remyelination capacity was lost, suggesting that Cystatin F expressed by microglia may play an important role in demyelination and remyelination. The study demonstrated that absence of the Cys F gene and the resulting disinhibition of cathepsin C aggravates the demyelination, and this finding may be related to the increased expression of the glia-derived chemokine, CXCL2, which may attract inflammatory cells to sites of myelin sheath damage [14]. The described mechanism is shown in Figure 3.

New studies employing Mendelian randomization techniques also link cathepsin gene polymorphisms to neurodegenerative diseases, including MS [61]. The involvement of cathepsins in MS pathogenesis underscores their role in immune regulation, demyelination, and neurodegeneration. Elevated levels of cathepsins B, S, L, and X/Z in MS lesions and immune cells, alongside reduced expression of their endogenous inhibitors such as cystatin F, suggest a disrupted protease–inhibitor balance, contributing to disease progression. These findings position cathepsins not only as markers of pathological activity but also as potential therapeutic targets. Emerging genetic and epigenetic evidence further supports their role in disease susceptibility and progression. Future research should focus on refining our understanding of the cathepsin–cystatin axis and its therapeutic modulation across different stages of MS.

## 8. Cathepsins and Huntington’s Disease

Huntington’s disease (HD) is a rare, autosomal-dominant neurodegenerative disease (4.88 of 100,000 patients) [115]. HD is affiliated with the *HTT* gene mutation, an expanded repetition of CAG trinucleotide, which contributes to the elongation of polyglutimine tract at the N-terminal of the protein. Mutated huntingtin (mhtt) is toxic and is inclined to cluster [116]. Studies show that polyQ fragments deposit in the neurons of HD patients due to a lack of degeneration by the proteasome [117]. This is because the disease manifests as a progressive motor dysfunction, similar to hyperkinesia and neuropsychiatric disorders, including depressed mood, disinhibition, euphoria, or aggression in 20–50% of patients resulting in premature death [118]. HD has a five-grade scale of severity (0–4) [119]. Moreover, some findings prove mhtt translation elevates levels of lysosomal proteases: cathepsin D, cathepsin L, cathepsin B, cathepsin X/Z and autophagy [110,120]. Cathepsins take part in the degradation of mhtt in the endolysosomal pathway, lysosomal activity may contribute to affecting cleavage products of N-terminal fragments known as A and B. These A and B fragments cluster in the inclusions of the nucleus and cytoplasm of the neurons [121].

**Figure 4:**
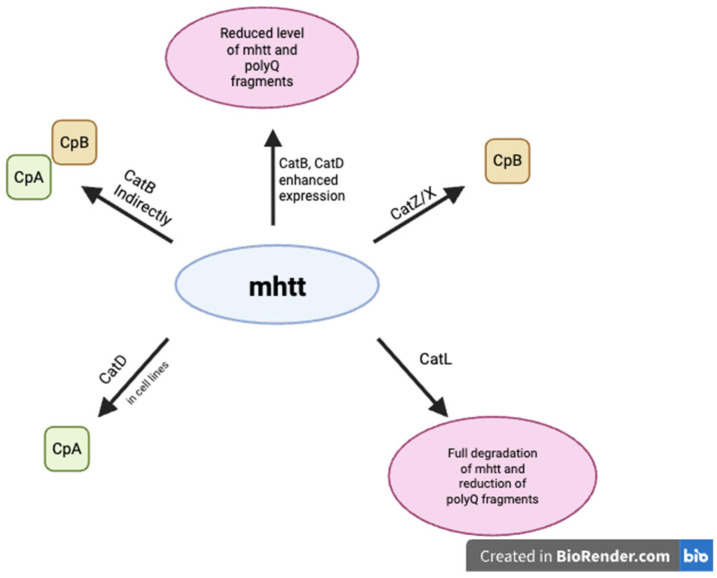
Involvement of cathepsins in Huntington’s disease pathogenesis.

Investigations in one study of Bhutani et al. [120] have shown the crucial role of cathepsins L and Z in the rapid degradation of extended polyglutamine sequences proteins and an important role in protection against the destructive aggregation of these proteins in mice brain models. End products of the degeneration of mhtt suggest that cathepsin L initiates the process as an endopeptidase, and then smaller products are cut by cathepsin Z as a carboxypeptidase. Moreover, inhibition of these cathepsins aggravates the deposition of the toxic polyQ protein. Other studies have shown the neuroprotective effect of elevated expression of cathepsins B and D due to decreased levels of full-length and fragmented mhtt in HEK cells. Furthermore, inhibition of both cathepsins B and D simultaneously aggravates mhtt neurotoxicity more than separately. This evidence indicates that both cathepsins take part in the degeneration of mhtt [122]. However Kim et al. [121] suggests that cathepsin D might contribute to disease progression as a consequence of creating N-mhtt fragments which are stable and potentially toxic. The involvement of cathepsins in HD pathogenesis is presented in Figure 4. There is insufficient evidence to unequivocally determine the exact role of cathepsins in Huntington’s disease. However, CatZ is highly likely to be responsible for cpB generation, reflecting current understanding. Cathepsin-based therapies may represent a future approach for the treatment of HD. The findings highlight the need for further research.

## 9. Cathepsins and Epilepsy

Epilepsy is a disorder of the brain, represented as a sustained inclination towards the generation of seizures. Epilepsy might be caused by the neuronal hyperactivity or synchronized neuronal activity, and is diagnosed when at least one episode with high risk of recurrence, or if two unprovoked seizures with temporary neurological symptoms occur (known as epileptic seizure in the International League Against Epilepsy (ILAE)) [123]. Status epilepticus is a state where seizures last at least 30 min [124]. Epilepsy is a ubiquitous disease that spreads globally; in 2021, there were 51.7 million people globally with this disorder [125]. The myoclonic epilepsy type, Unverricht–Lundborg (EPM1), is caused by a loss-of-function mutation of cystatin B, an inhibitor of cysteine proteases known as cathepsins [126,127].

One study suggested that the dysregulation of cystatin B–cathepsin B signaling might be crucial for protecting neurons from degeneration and death due to the oxidative stress in EPM1. Cystatin B-deficient mice had accumulated oxidative neuronal damage expressed as reduced antioxidant capacity and increased sensitivity to lipid peroxidation [128]. Evidence shows that cathepsin B gene *CTSB* knockout decreased neuronal granule cell damage in mice models [129]. Removal of cathepsins L and S in cystatin B-deficient mice improved no aspect of EPM1, whereas cathepsin B removal resulted in a 36–89% reduction in granule cell apoptosis [130]. Additionally, in rat models succeeding 40 rapidly recurring seizures evoked by hippocampal kindling stimulations, an inclined synthesis level of cystatin B in forebrain neurons was found [131]. Considering this inhibiting cathepsin B in EPM1 might be a potential therapy strategy. Studies on rats have shown that status epilepticus triggers within 60 min after the episode of nuclear translocation of mitochondrial proteins and lysosomal enzymes including cathepsins B and D [132]. Dynamics of cathepsin expression in rats after epileptic seizure differs over time. Cathepsin K has its peak expression 1 day after an epileptic seizure episode and stays increased in the chronic phase. However, robust expression of the cathepsins B, D, and L occurs 1 week after an epileptic episode which correlates with microglial activation [133]. Further studies of this complex mechanism are needed since the exact role of cathepsins in epilepsy is not yet known. However, studies on animal models have shown that lysosomal proteases play an important role in neurodegeneration. The role of cathepsins in epilepsy is shown in Table 4. Targeting cathepsins could represent a potential therapeutic approach in the future.

## 10. Cathepsins and Amyotrophic Lateral Sclerosis

Amyotrophic lateral sclerosis (ALS) is a rare, neurodegenerative disease of the CNS, characterized by progressive degeneration of upper and lower motor neurons, leading to muscle weakness and atrophy, and ultimately progressing to respiratory failure and death [134,135]. ALS can manifest as a sporadic form (90–95%), typically with later onset, or as a familial form (fALS), which presents at a younger age and accounts for 5–10% of cases [135,136]. The familial form is associated with genetic mutations, including SOD1, C9orf72, FUS, and TARDBP [137]. Comprehensive meta-analyses have revealed regional differences in ALS incidence, with the highest rates observed in Western Europe and North America. Gender differences have also been noted, with men being significantly more affected than women (male-to-female ratio 1 between 2) [136,138].

Depending on the disease phenotype, it may begin with bulbar-onset ALS, involving dysphagia (swallowing difficulties) and dysarthria (speech impairment), or with the more common classical limb-onset ALS, characterized by initial involvement of the lower limbs and gradual spread to other regions of the body. A subset of patients also exhibit cognitive dysfunction, often presenting as frontotemporal dementia [134,135,137,139]. Despite ongoing progress in understanding the molecular mechanisms underlying ALS, current treatment options remain symptomatic only, and no effective disease-modifying therapy has been developed to date [135]. Therefore, a key focus of contemporary research is the identification of novel therapeutic targets for molecular engineering strategies aimed at slowing or halting neurodegeneration. In this context, increasing interest has been directed toward cathepsins, whose roles in neurodegenerative processes are still being elucidated, with evidence indicating their dysregulated activity in ALS. Scientific reviews have revealed significant variability in the expression of cathepsins B, C, D, H, and X/Z within the CNS and suggest their potential involvement in neurodegenerative processes through the induction of inflammatory responses and degradation of pathological proteins [110]. In the case of cathepsin B, increased expression has been observed in neuroinflammatory processes in ALS patients as well as in animal models. High levels of this enzyme have been detected postmortem in the anterior horns of the spinal cord, suggesting it may play a crucial role in motor neuron degeneration. Concurrently, increased expression of its endogenous inhibitor, cystatin C, has been noted, potentially mitigating excessive autophagy and the neurotoxic overactivity of cathepsin B [110,140]. However, Mendelian randomization studies have not demonstrated a significant association between cathepsin B overexpression and ALS risk, suggesting that elevated levels may be a secondary consequence of neurodegeneration rather than a primary cause [35,61]. Cathepsin D has been implicated in the regulation of autophagy in ALS. Huang et al. showed that the long non-coding RNA lnc-HIBADH-4, which is downregulated in ALS patients, modulates cathepsin D activity. The dysregulation of this autophagy–lysosome pathway results in the accumulation of pathological proteins such as TDP-43, a hallmark of ALS pathology. This positions cathepsin D activators as potential therapeutic targets for ALS patients [141]. Cathepsin H has been shown by Kibinge et al. [142] to amplify inflammatory responses in microglial cells. They also demonstrated that this is driven by increased expression of the CTSH gene, which encodes cathepsin H in ALS patients. Notably, CTSH does not exhibit pleiotropy, i.e., it does not affect other phenotypes, making it a promising therapeutic target through the suppression of its expression to reduce neuroinflammatory responses that underpin ALS pathophysiology [7,142]. Cathepsin X has been found to have a detrimental effect on microglial activation and degradation of signaling proteins, leading to cytoskeletal dysfunction and motor neuron degeneration [110]. However, similar to cathepsin B, Mendelian randomization studies have not confirmed a causal role for cathepsin X in ALS [35,61,110]. The dysregulated activity of cathepsins in ALS highlights their potential involvement in key pathogenic mechanisms, including neuroinflammation, impaired autophagy, and protein aggregation. While elevated expression of cathepsins B, D, H, and X has been associated with motor neuron degeneration and glial activation, genetic evidence does not consistently support a primary causal role. Nonetheless, specific cathepsins—particularly D and H—exhibit functional relevance and molecular specificity that position them as promising therapeutic targets. Further investigation into the cathepsin–cystatin balance, along with exploration of lncRNA and gene regulatory pathways, may yield novel strategies for modifying disease progression in ALS; the therapeutic potential of cathepsins in ALS is summarized in Table 5.

## 11. Clinical Use of Cathepsins

Cathepsins and their endogenous inhibitors have become the focus of preclinical and translational research due to their involvement in diverse pathological processes. Although their use in clinical settings remains investigational, emerging evidence supports their potential as therapeutic targets and disease biomarkers. One area of promising application is traumatic brain injury (TBI), a condition in which cathepsin B is markedly upregulated in both animal models and human patients. Preclinical studies have demonstrated that pharmacological inhibition of cathepsin B significantly ameliorates neurobehavioral deficits following TBI. Notably, genetic knockout of cathepsin B in murine models results in significant attenuation of injury-related impairments, highlighting its pathogenic relevance. Among available inhibitors, E64d, a broad-spectrum cysteine protease inhibitor with specificity for cathepsin B, has shown efficacy in experimental TBI models and has been deemed safe for human use. These findings collectively provide a compelling rationale for advancing cathepsin B inhibitors toward clinical development and evaluation [143]. Comparable therapeutic effects have been observed following cathepsin S inhibition in TBI. Expression of cathepsin S increases as early as one hour post-injury, indicating its involvement in the acute phase of trauma-induced neuroinflammation. In murine models, pharmacological inhibition of cathepsin S leads to significant reductions in neuroinflammatory cytokine levels and cerebral edema, yielding functional improvements similar to those observed with cathepsin B blockade [144]. Clinical translation of cathepsin S inhibition has also begun. A phase I clinical trial evaluating a selective cathepsin S inhibitor in healthy volunteers (*n* = 21) reported transient reductions in plasma cathepsin S activity, along with favorable safety and tolerability profiles [145]. While this pharmacodynamic effect supports further development, therapeutic efficacy in disease contexts remains to be established. A randomized, double-blind, placebo-controlled trial assessing the same compound in patients with primary Sjögren’s syndrome, a chronic autoimmune condition, failed to demonstrate significant clinical benefits [146]. This discrepancy may reflect several intrinsic limitations of cathepsin-targeted strategies: functional redundancy among cathepsin isoforms; lack of disease specificity, as many cathepsins are elevated across disparate pathological states; broad physiological roles that complicate selective inhibition; and challenges related to effective delivery across the blood–brain barrier. Beyond TBI, ischemic stroke represents another promising indication for cathepsin-targeted diagnostics and therapeutics. A recent human study reported that plasma cathepsin L levels were significantly elevated in acute ischemic stroke patients compared to healthy controls, suggesting a potential biomarker role [147]. In preclinical stroke models, pharmacological inhibition of cathepsin L reduced infarct volume and improved neurobehavioral outcomes, though it remains unclear whether cathepsin L actively contributes to infarct expansion or simply reflects secondary inflammatory processes. Intriguingly, modulation of circulating cathepsin L was sufficient to improve outcomes in experimental models, underscoring the systemic relevance of this enzyme. Outside the nervous system, cathepsins have also been investigated in cardiovascular disease. In a model of dilated cardiomyopathy, elevated levels of cathepsin B and cathepsin L were found to correlate with the severity of left ventricular dysfunction. These proteases may thus serve as circulating biomarkers for cardiac remodeling and functional decline [148].

In summary, while cathepsins have yet to enter routine clinical practice, their pathophysiological relevance, accessibility in biofluids, and modifiability via small-molecule inhibitors make them attractive candidates for biomarker development and therapeutic intervention. However, successful translation will require overcoming key challenges related to specificity, redundancy, and CNS delivery. A summary of all potential application of cathepsins is presented in Table 6.

## 12. Conclusions and Future Directions

Despite increasing interest and progress in this field, many questions remain unanswered. The context-dependent functions of cathepsins, their interactions with endogenous inhibitors like cystatins, and the regulatory networks that govern their expression and activity in the CNS are still not fully elucidated. Moreover, inconsistencies between experimental models and human data underscore the need for more translationally relevant approaches. The potential for cathepsins to serve as diagnostic biomarkers, prognostic indicators, and therapeutic targets remains an exciting but underexplored avenue. Future research should aim to decipher molecular mechanisms driving cathepsin-mediated neurotoxicity versus neuroprotection, investigate cathepsin–inhibitor balance (including the role of cystatins and the therapeutic modulation of this axis), and develop selective cathepsin modulators, assessing their therapeutic potential in preclinical and clinical settings. In conclusion, cathepsins represent a promising but complex class of molecular players in the pathogenesis of neurological diseases. Their further study may not only deepen our understanding of disease mechanisms but also pave the way for novel diagnostic and therapeutic strategies in neurology.

## Figures and Tables

**Table 1:** Key characteristics and associations of cathepsins with neurological diseases.

Cathepsin	Key Characteristics	Role in CNS	Disease Associations
D	Aspartic protease; degrades α-synuclein	Protein clearance; overexpression causes neuronal death	Parkinson’s disease, Alzheimer’s disease
H	Dual exo- and endopeptidase; in microglia	Modulates immune response; promotes proinflammatory cytokines and neuronal damage	Neuroinflammation
B and L	Cysteine proteases; intracellular proteolysis and ECM remodeling	Maintain brain integrity; involved in cholesterol trafficking	Niemann–Pick type C-like pathology
X	Carboxypeptidase; expressed in monocytes/macrophages	Mediates microglial activation and neurotoxicity; inhibition reduces inflammatory markers	Neuroinflammatory conditions
C	Activates serine proteases; low brain expression	Induced by inflammation in microglia; role in neuroinflammation unclear	Demyelination
S	Active at neutral pH	Involved in aging and neurodegeneration; elevated in ALS models	Aging, ALS
K	Known for bone resorption; also in brain parenchyma	Influences behavior, learning, and memory	Neurobehavioral regulation
Cystatin C	Endogenous inhibitor of cathepsins	Regulates cathepsin activity; imbalance linked to impaired remyelination	Multiple sclerosis, Alzheimer’s disease

**Table 2:** Influence of cathepsins on PD pathophysiology.

Cathepsin	Influence	References
B	degradation of α-synuclein deficiency linked with brain atrophy, neurodegeneration, and reactive astrocytosis inhibition disturbs autophagy, hinders pre-formed α-synuclein fibrils clearance, impairs glucocerebrosidase function, and contributes to lysosomal content deposition activity may be altered by *LRRK2* mutation knockdown decreases fibril-induced aggregates formation impacts the synthesis and release of interleukin-1β	[11,19,22,31,35,36]
D	degradation of α-synuclein restores autophagy and endo-lysosome functions neuroprotective effect via interplay with calcineurin induces α-synuclein truncations oligomerization and downstream pore formation knockdown contributes to a decrease in inflammation-mediated dopaminergic neurodegeneration via inhibition of the NF-κB signaling pathway	[8,11,13,23,42]
L	degradation of α-synuclein deficiency linked with brain atrophy, neurodegeneration, and reactive astrocytosis activity may be altered by *LRRK2* mutation inhibition restores decreased glucocerebrosidase levels and diminishes phosphorylated α-synuclein burden	[11,19,31,43]
X	induces microglia activation-mediated neurodegeneration inhibitor exhibits neuroprotective effects	[39,40,41]

**Table 3:** Significance of cathepsins in stroke management.

Cathepsin	Pathological Role	Key Mechanism	Significance in Stroke Management
C	Aggravates neuroinflammation and neurotoxic microglial polarization	Activates inflammatory signaling pathways	Potential biomarker; inhibitor may reduce inflammation
S	Amplifies stroke related injury	BBB disruption, vascular leakage, neuronal injury	Potential biomarker candidate; inhibition reduces infarct size
B	Promotes ferroptosis, exacerbates aneurysm progression	Iron-dependent cell death	Inhibition mimics ferroptosis blockade; potential therapeutic target
K	Prevention of hemorrhagic transformation following rtPA	Extracellular matrix degradation	Potential therapeutic target for rtPA complication
E	Increased ischemic stroke risk	Chronic neuroinflammation and brain injury, development of atherosclerotic plaques	Emerging biomarker candidate
O	Increased ischemic stroke risk	Development of atherosclerosis	Emerging biomarker candidate
G	Indirect stroke risk via prothrombotic state found in COVID-19 patients	Neutrophil activation, protease activity	Potential biomarker candidate
D	Prevention of neuron cell death following protein accumulation in stroke	Supports lysosomal function, prevents protein accumulation	Neuroprotective therapeutic target
A	Genetic small vessel disease with stroke and leukoencephalopathy	Endothelin-1 accumulation impairs myelination	Promising gene therapy in models

**Table 4:** Cathepsins’ role in epilepsy.

Cathepsin	Key Findings	Role in Epilepsy
B	Knockout reduces neuronal apoptosis in EPM1; elevated after seizures	Major contributor to neurodegeneration
D	Nuclear translocation within 60 min post-status epilepticus	Early mediator of neuronal injury
L	Increased 1 week post-seizure; knockout ineffective in EPM1	Possibly involved in late inflammation
S	Knockout does not reduce EPM1 pathology	Minor or redundant role
K	Peaks 1 day after seizures; remains elevated	May support chronic damage progression
Cystatin B	Deficiency causes oxidative stress; upregulated after seizures	Key endogenous cathepsin inhibitor

**Table 5:** Therapeutic potential of cathepsins in ALS.

Cathepsin	Expression in ALS	Proposed Role	Therapeutic Potential
B	↑ in spinal anterior horns (postmortem)	Motor neuron degeneration, neuroinflammation	Possible target, but likely secondary to neurodegeneration
D	Dysregulated via lncRNA (lnc-HIBADH-4)	Autophagy regulation, TDP-43 clearance	Activators may restore autophagic flux
H	↑ CTSH gene expression in ALS	Amplifies microglial inflammation	Gene suppression may reduce neuroinflammation
X/Z	↑ in ALS models	Microglial activation, cytoskeletal dysfunction	Potential target; needs more validation
C	Poorly characterized	Neuroimmune modulation (hypothesized)	Requires further study
Cystatin C	↑ in ALS tissues	Counteracts cathepsin B, modulates autophagy	Protective role; potential biomarker

**Table 6:** Role of cathepsins in pathogenesis of neurological diseases.

Cathepsin	Protease Type	Neurological Role/Application
Cathepsin A	Serine carboxypeptidase	Associated with CARASAL, a hereditary stroke and leukoencephalopathy syndrome involving cerebral small vessel disease.
Cathepsin B	Cysteine protease	Acts as a biomarker and therapeutic target in Alzheimer’s disease (AD), Parkinson’s disease (PD), and epilepsy; degrades amyloid-beta and α-synuclein; contributes to neuroinflammation.
Cathepsin C	Cysteine protease	Enhances M1 polarization and demyelination in multiple sclerosis (MS); observed in stroke and neuroinflammation models; potential therapeutic target.
Cathepsin D	Aspartic protease	Major lysosomal enzyme involved in α-synuclein degradation in PD; elevated in AD and dementia; biomarker and potential therapeutic agent in stroke and lysosomal storage disorders.
Cathepsin E	Aspartic protease	Involved in neuroinflammation and amyloid-beta production in AD; therapeutic target in aging-related neurodegeneration.
Cathepsin F	Cysteine protease	Studied for potential role in neurodegeneration; precise neurological significance remains unclear.
Cathepsin G	Serine protease	Promotes platelet aggregation and vascular pathology; may contribute to stroke pathogenesis.
Cathepsin H	Cysteine protease	Expressed in perivascular microglia; promotes inflammatory cytokine release; implicated in PD, ALS, and other neuroinflammatory disorders.
Cathepsin K	Cysteine protease	Linked to behavioral deficits and anxiety; knockout studies show learning/memory impairment; contributes to stroke pathology.
Cathepsin L	Cysteine protease	Crucial for lysosomal function; involved in AD, PD, and Huntington’s disease (HD); regulates autophagy and neuronal survival.
Cathepsin O	Cysteine protease	Genetic associations suggest involvement in stroke susceptibility; more studies are needed to clarify function in CNS.
Cathepsin S	Cysteine protease	Contributes to chronic inflammation in AD, MS, ALS, and stroke; potential biomarker and drug target.
Cathepsin V	Cysteine protease	Limited data in CNS context; included in cathepsin family profiling studies.
Cathepsin W	Cysteine protease	Limited data in neurology; role in immune regulation and potential neuroinflammatory processes hypothesized.
Cathepsin X/Z	Cysteine protease	Key regulator in microglial activity; implicated in PD, MS, HD, and neurodegenerative inflammation; therapeutic potential shown in EAE and toxin models.

## Data Availability

Not applicable.
